# Long-term outcome and health-related quality of life in difficult-to-wean patients with and without ventilator dependency at ICU discharge: a retrospective cohort study

**DOI:** 10.1186/s12890-016-0295-0

**Published:** 2016-09-27

**Authors:** P. Depuydt, S. Oeyen, S. De Smet, S. De Raedt, D. Benoit, J. Decruyenaere, E. Derom

**Affiliations:** 1 Intensive Care Department, Ghent University Hospital, De Pintelaan 185, Ghent, 9000 Belgium; 2 Ghent University, Sint-Pietersnieuwstraat 10, Ghent, 9000 Belgium; 3 Department of Respiratory Diseases, Ghent University Hospital, De Pintelaan 185, Ghent, 9000 Belgium; 4 Heymans Institute of Pharmacology, Ghent University Hospital, De Pintelaan 185, Ghent, 9000 Belgium

**Keywords:** Prolonged mechanical ventilation, Quality-of-life, Tracheostomy

## Abstract

**Background:**

Long-term outcome and quality of life (QOL) in patients requiring prolonged mechanical ventilation after failure to wean in the ICU is scarcely documented. We aimed to evaluate long-term survival and QOL in patients discharged from the ICU with a tracheostomy for difficult weaning, and with or without ventilator dependency at ICU discharge.

**Methods:**

We retrospectively investigated post-ICU trajectories and survival in patients requiring tracheostomy for difficult weaning admitted to the medical ICU of a tertiary center between 1999 and 2013, discriminating between patients who were ventilator dependent or were weaned at ICU discharge. In 2014, a QOL assessment was done in survivors with the use of the Short Form Health Survey (SF-36) and the Severe Respiratory Insufficiency questionnaire.

**Results:**

A total of 114 patients was included, of whom 59 were ventilator dependent and 55 were weaned at ICU discharge. One-year survival rates were 73 % and 69 %, respectively. Overall QOL scores for physical functioning were low, and not significantly different between patients ventilated and those weaned at ICU discharge; scores for social functioning and mental health were less below norm and similar between both groups.

**Conclusions:**

Long-term survival in patients discharged from the ICU with tracheostomy and ventilator dependency after failure to wean was not significantly different from that of patients with tracheostomy and weaned at ICU discharge. Despite the physical QOL scores being low in both groups, mental QOL was acceptable. Given the intrinsic limitations of this retrospective study, prospective and preferentially multicenter studies are required to confirm these preliminary results.

## Background

Mechanical ventilation following endotracheal intubation is a potentially life-saving intervention in patients with acute respiratory failure (ARF). However, in an increasing number of patients who survive the acute phase of critical illness, subsequent weaning from mechanical ventilation is difficult, prolonged or may ultimately prove to be impossible [1].

In difficult-to-wean patients, it is widespread practice to place a tracheostomy tube to facilitate weaning, decrease complications associated with translaryngeal intubation and increase patient comfort, although conclusive evidence for these benefits is lacking [2, 3]. Despite this intervention, patients may remain ventilator dependent due to pre-existing neuromuscular or respiratory illness, sequelae of the ARF event, concomitant cardiac dysfunction, persistent paralysis or intensive care unit (ICU)-acquired neuromuscular weakness, or a combination of these factors [4].

In patients who recover sufficiently to survive without the close monitoring and supportive care provided in the ICU, prolonging invasive mechanical ventilation outside the ICU may be considered; a decision to embark on this trajectory usually involves shared decision making between the patient, his caregivers, and the ICU and pulmonology department medical teams. However, the outcome and, especially, the quality of life (QOL) of these patients following ICU discharge is as yet poorly known. Yet, this information is essential to evaluate the benefits and downsides of this complex and costly care [5].

## Methods

This single-center retrospective study, conducted at Ghent University Hospital, reports on the post-ICU trajectory, long-term outcome and QOL in two cohorts of patients that were discharged from the ICU with placement of a tracheostomy because of difficult weaning: the first cohort was still dependent on mechanical ventilation at ICU discharge, while the second cohort was weaned in the ICU. All patients were admitted to the Medical Intensive Care Unit (MICU) and were subsequently discharged to the Department of Respiratory Medicine. The 14-bed MICU is part of a 66-bed ICU, which also includes a surgical (22 beds), cardiosurgical (10 beds) and a pediatric ICU (14 beds) and a burn unit (6 beds). The MICU admits critically ill patients of at least 15 years old and also serves as a regional referral center for difficult-to-wean patients. Nurse to patient ratio is 1:2. The MICU yearly admits 700 to 800 patients, of whom 40 % require mechanical ventilation; 15 % receive mechanical ventilation for >7 days. The Department of Respiratory Medicine includes a center for home mechanical ventilation in charge of 100 patients; in 2014, chronic home ventilation was initiated in 19 patients, in whom 3 required invasive ventilation (i.e. with tracheostomy).

Patients who were admitted to the MICU between 1999 and 2013 were eligible for inclusion in the retrospective cohort if they had received a tracheostomy for difficult weaning during their ICU stay and were discharged to the Department of Respiratory Medicine while this tracheostomy was still present. Patients with neuromuscular or pulmonary disease in whom chronic mechanical ventilation was initiated semi-electively were not included. They were classified as ventilated patients if they were still dependent on mechanical ventilation for at least part of the day at ICU discharge, and as weaned patients if they did not require mechanical ventilation for at least 72 h at ICU discharge.

From the medical and administrative records we collected demographics, comorbidities, admission diagnosis, etiology of acute respiratory failure and whether patients were admitted through the emergency department or were referred from other hospitals. Charlson comorbidity scores were calculated to weight comorbidities [6]. We recorded trajectories following ICU discharge and vital status at hospital discharge. In hospital survivors, we noted whether patients still had a tracheostomy and whether or not they still received mechanical ventilation (including non-invasive ventilation) at the time of hospital discharge or thereafter.

We collected survival data at the end of 2014 by consulting medical and administrative patient records and by contacting the patient’s general or referring physician if these records were uninformative about the patient’s vital status. Between June and December 2014, all surviving patients were invited by phone to invite them to participate in the QOL study, which was done by means of the Medical Outcomes Study 36-item Short Form Health Survey (SF-36) and the Severe Respiratory Insufficiency (SRI) questionnaire. Both surveys were completed as paper forms sent by mail. The SF-36 questionnaire consists of 36 items measuring eight multi-item domains in a 0–100 scale, a higher score representing a better condition: physical (PF) and social functioning (SF), role limitations due to physical (RP) or emotional problems (RE), mental health (MH), vitality (VT), bodily pain (BP) and general health (GH) [7, 8]. The reliability and validity of the SF-36 has been evaluated in the critically ill population [9].

The SRI questionnaire has been developed to assess health-related QOL in patients with severe chronic respiratory failure resulting from a broad spectrum of underlying disorders. The SRI questionnaire contains 49 questions in 7 domains: respiratory, physical functioning, sleep quality, social functioning, feelings of fear, mental health and social functioning; a summary score is calculated over all domains. The SRI has been validated for a broad spectrum of patients with chronic respiratory failure [10], in particular those patients requiring home non-invasive mechanical ventilation [11].

The study was approved by the local ethical committee (Ghent University Hospital Ethical Committee). For the retrospective assessment, informed consent was waived, but from patients who underwent the QOL assessment, a signed informed consent was obtained.

Binary and categorical variables are presented as frequencies and percentages. Numerical variables are presented as mean (with standard deviation) or as median (with interquartile range) if normally, respectively non-normally distributed. To compare numerical values, the Student’s T-test or Mann–Whitney-U test were used depending on the variable distribution; for categorical variables, the Fisher Exact test was used. Kaplan-Meier survival probabilities were calculated and were compared using the log-rank test. All reported p-values are two-tailed and a cut-off level of 0.05 was used to conclude for significance. All statistical analysis was performed using SPSS 22.0 software.

## Results

**Fig. 1:**
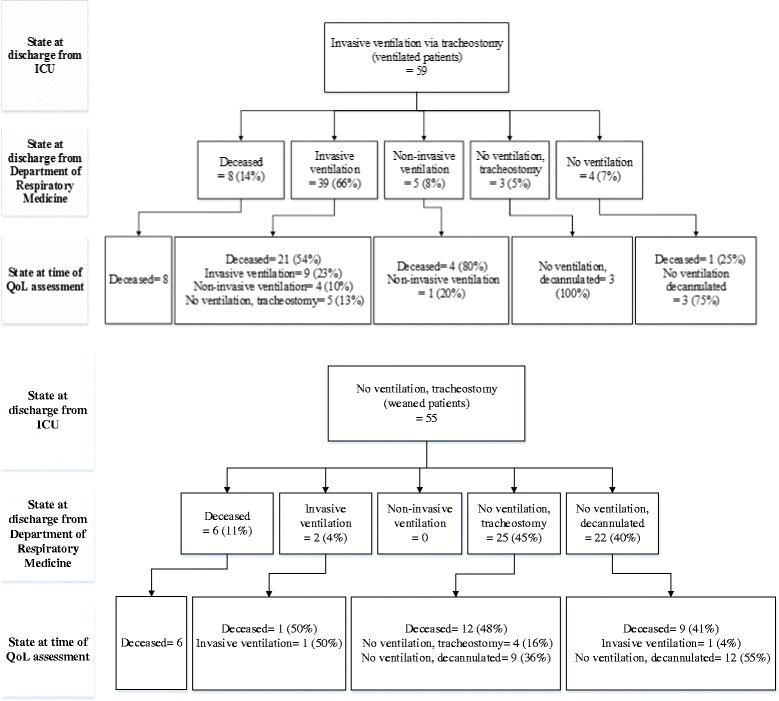
Patient trajectories following ICU discharge

A total of 114 patients with a tracheostomy at ICU discharge, of whom 59 still were ventilator dependent and 55 were weaned, were included for survival analysis. This cohort represents 1 % of all patients admitted to the MICU, and 7 % of all MICU patients requiring tracheostomy during the study period. Patient characteristics are detailed in Table 1. Ventilator-dependent patients had more frequently underlying neuromuscular disease (19 % vs. 4 %, *p =* 0.02), and had less often pneumonia (32 % vs. 67 %, *p <* 0.001) and septic shock (2 % vs. 22 %, *p <* 0.001) as primary cause of acute respiratory failure as compared with weaned patients. A similar number of ventilator-dependent and weaned patients (58 %) were referrals from other hospitals. Timing and indication of tracheostomy and length-of-stay at the ICU were not significantly different between both groups. Length-of-stay at the pulmonology department was significantly longer for ventilator-dependent patients vs. weaned patients (46d vs. 29d, *p =* 0.004) but hospital mortality was similar in both groups. Patient trajectories following ICU discharge are detailed in Fig. 1. Twelve ventilator-dependent patients were subsequently weaned (7 during the same hospitalization episode and 5 at a later date) and nine patients were converted to non-invasive ventilation (5 during the same hospitalization episode and 4 at a later date). Two patients who were weaned at ICU discharge again required ventilator support and tracheostomy at hospital discharge (1 patient) or at a later date (1 patient). Ventilator-dependent patients at ICU discharge were more frequently discharged at home directly as compared to weaned patients (45 % vs. 27 %, *p =* 0.05). The number of readmissions following hospital discharge was not significantly different between both groups.

**Table 1:** Patient characteristics

	Ventilated patients (*n =* 59)	Weaned patients (*n =* 55)	*p*-value
Age (years)	60 (53–70)	62 (53–62)	0.43
Male gender	35 (59)	34 (62)	0.78
Comorbidities			
Smoker	18 (35)	21 (47)	0.23
Heavy drinker	11 (19)	16 (30)	0.17
Obesity	12 (20)	10 (18)	0.78
Congestive heart failure	16 (27)	12 (22)	0.51
Peripheral vascular disease	10 (17)	9 (17)	0.93
Cerebrovascular disease	3 (5)	6 (11)	0.31
Diabetes	13 (22)	12 (22)	0.68
Chronic kidney disease	6 (10)	6 (11)	0.89
Chronic liver disease	2 (3)	6 (11)	0.02
Malignancy^a^	7 (12)	12 (22)	0.15
COPD	20 (34)	23 (42)	0.71
Restrictive lung disease	5 (8)	7 (13)	0.92
Obstructive sleep apnea syndrome	8 (14)	5 (9)	0.45
Neuromuscular disease	11 (19)	2 (4)	0.02
Charlson Index of Comorbidity	5 (4–7)	6 (4–8)	0.16
Main admission diagnosis^b^			
Postoperative	6 (10)	6 (11)	0.92
Acute tetraplegia	7 (12)	8 (15)	0.79
Trauma (excluding tetraplegia)	3 (5)	4 (7)	0.71
Acute neurologic failure (excluding tetraplegia)	10 (17)	1 (2)	0.06
Acute cardiac failure	6 (10)	4 (7)	0.74
Septic shock	1 (2)	12 (22)	<0.001
Pneumonia (without septic shock)	19 (32)	37 (67)	<0.001
Exacerbation of COPD or other structural lung disease	16 (27)	10 (18)	0.26
Acute on chronic hypercapnic failure at admission^c^	22 (37)	12 (22)	0.07
Vasopressor therapy during ICU stay	27 (46)	35 (63)	0.48
Hemodialysis during ICU stay	9 (15)	10 (18)	0.67
Time to tracheostomy (days)	14 (8–20)	16 (10–25)	0.23
Time to ICU discharge (days)	20 (10–39)	29 (15–51)	0.17
Time to weaning (days)	-	18 (7–31)	-

Data are reported as numbers (%) or median (interquartile range)

^a^Malignancy was considered cured or in remission at ICU admission in all patients

^b^One patient may have multiple admission diagnoses

^c^As evidence by partially compensated respiratory acidosis and underlying pulmonary or extrapulmonary disease associated with respiratory pump failure

**Fig. 2:**
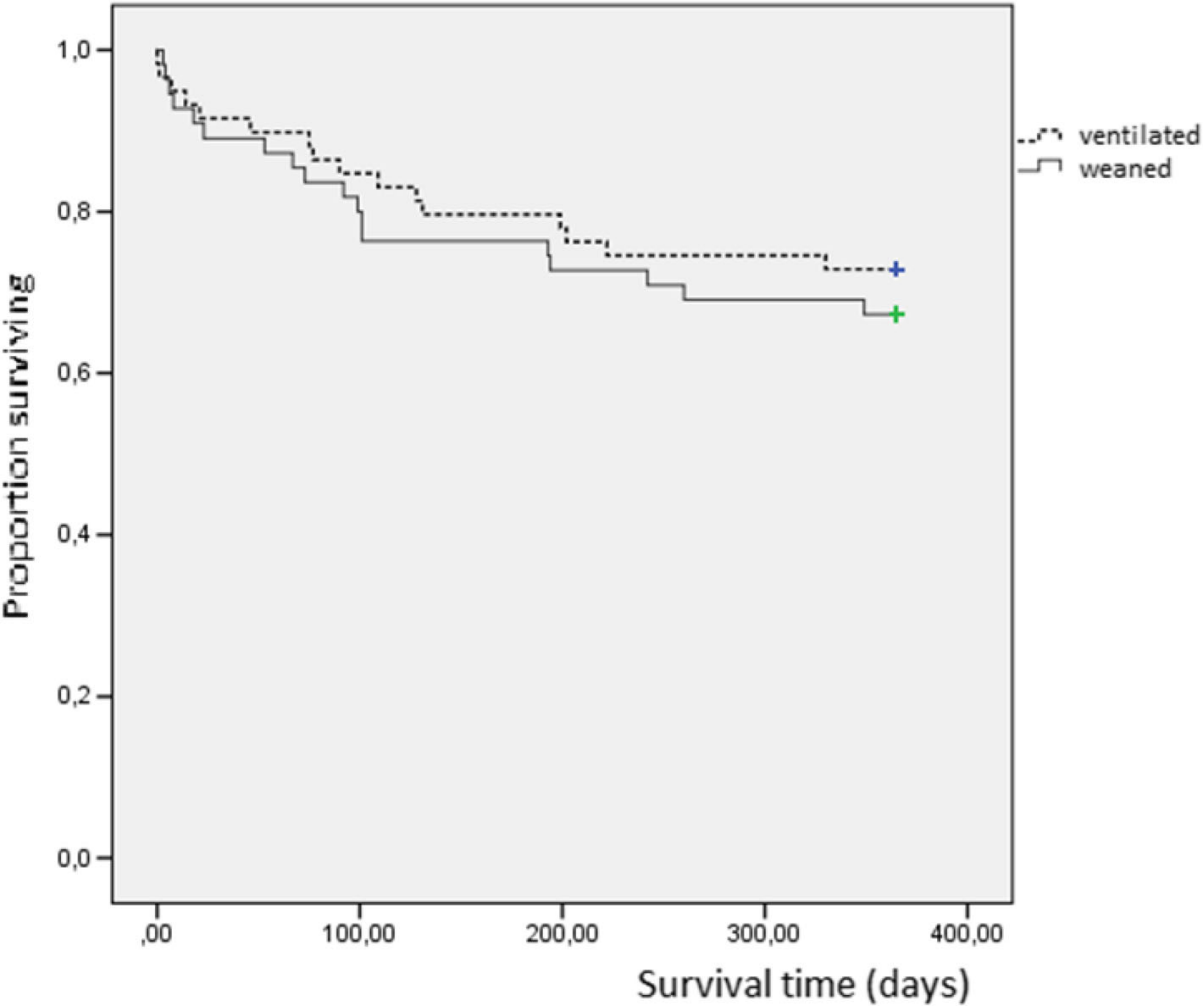
Survival curves of patients with tracheostomy who were ventilator-dependent (dashed line) (*n =* 59) or who were weaned (full line) (*n =* 55) at ICU discharge

One-year survival of patients with ventilator dependency or weaned at ICU discharge was 73 % and 69 % respectively (*p =* NS); Kaplan-Meier survival curves are shown in Fig. 2. Charlson comorbidity index was significantly associated with survival in ventilator-dependent patients (*p =* 0.012) but not in weaned patients. When individual comorbidities were analyzed, chronic renal failure (*p <* 0.001) and cerebrovascular disease (*p =* 0.05) were negatively associated with survival. Admission diagnosis and etiology of acute respiratory failure were not related to survival. In patients with a follow-up of at least five years, 5-years survival rates were 40 % (16/40) for ventilator-dependent and 42 % (14/33) for weaned patients, respectively.

**Fig. 3:**
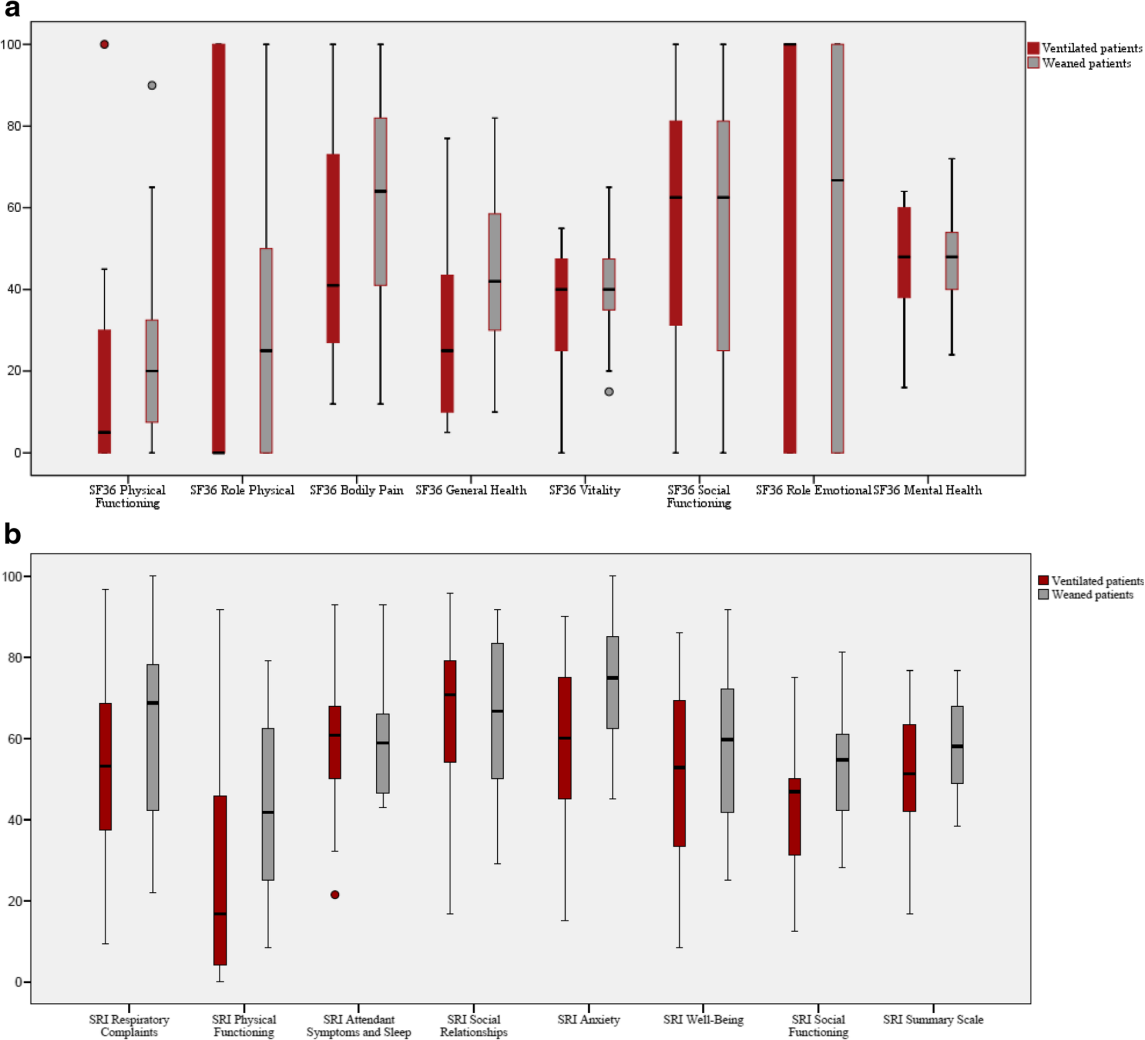
Quality-of-life as measured by the SF-36 questionnaire (**a**) and the SRI questionnaire (**b**) in patients who were discharged from the ICU with a tracheostomy (*n =* 43) and ventilator dependency (*n =* 21) or weaned (*n =* 22) at ICU discharge*. *In the group of patients with ventilator dependency at ICU discharge (*n*=21) and at time of QOL assessment, 7 patients still required invasive ventilation, 4 non-invasive ventilation, 1 patient had a tracheostomy without ventilation and 9 patients had no ventilation and no tracheostomy. In the group of patients who were weaned at ICU discharge (*n*=22) and at time of QOL assessment, 1 required invasive ventilation, 3 patients had a tracheostomy without ventilation, and 18 had no ventilation and no tracheostomy

QOL was assessed in 21 patients with ventilator dependency at ICU discharge after a median of 58 (24–103) months and in 22 weaned patients after a median of 52 (40–73) months. The overall response rate was 82,5 %; 4 patients could not be contacted, 2 had moved to an address abroad and 3 refused to participate. The 0–100 scores of the SF-36 and SRI are detailed in Fig. 3. Overall, QOL was low. SF-36 scores in the physical domains were low but not different between patients with ventilator dependency or weaned at ICU discharge. GH was significantly lower in ventilator-dependent vs. weaned patients(*p =* 0.04). The SF-36 scores in the more mental domains were better without significant differences between ventilator-dependent and weaned patients.

SRI showed overall lower QOL in ventilator-dependent vs. weaned patients (50 vs. 59, *p =* 0.04), with lower scores for physical functioning and feelings of fear, but with similar scores in social functioning, relations and mental health.

## Discussion

Deciding whether or not to embark for prolonged mechanical ventilation in critically ill patients with failure to wean in the ICU is complex, with many uncertainties regarding long-term outcome. In this monocentric study, survival of patients who were discharged from the ICU with tracheostomy and ventilator dependency was 73 % at one year and 40 % at five years. As compared to tracheotomized patients who were weaned at ICU discharge, patients with ventilator dependency had comparable survival, lower QOL in terms of physical functioning, but similar QOL in terms of social and emotional functioning.

Our findings show that at least a selection of patients with failure to wean can be offered a meaningful survival with an acceptable QOL in the longer term, despite the fact that the majority of them remain dependent on ventilator support. In our practice, patients are considered eligible for prolonged mechanical ventilation on an individual basis and after careful interdisciplinary consultation and using shared decision involving patients and caregivers. A prerequisite for prolonging mechanical ventilation outside the ICU is a reasonable life expectancy with a stable or only slowly evolving underlying illness, and absent or minimal extra-pulmonary organ dysfunction. Additional important criteria are good social support and an acceptable QOL before the acute event (as estimated after history-taking). There must be willingness to proceed on a prolonged medical trajectory with important patient and caregiver’s involvement, including revalidation, education and self-care. Recently, a decision aid has been developed for relatives of patients with prolonged mechanical ventilation, in which the goal of treatment was presented as a continuum of options ranging from maximizing life duration to maximizing comfort. Testing this aid in a before-after study revealed better concordance between physician and patient caregivers and greater comprehension. While this aid was essentially developed to assist surrogates of patients to understand and explore the patient’s values and preferences, it could also facilitate shared decision-making involving the patient himself: this requires however further study [12].

The only data that have been published regarding outcome of patients requiring post-ICU mechanical ventilation come from long-term acute care (LTAC) hospitals in the USA. In a large multicenter study of 1419 patients referred to 23 long-term care hospitals requiring post-ICU ventilation (the Ventilation Outcomes Study) [13, 14], survival at discharge was 75 % (86 % in our study); 72 % of patients discharged alive were weaned (14 % in our study) but only 29 % of survivors were discharged to home (45 % in our study), the remaining being transferred to other healthcare-facilities. In addition, 1-year survival following LTAC admission was only 30 % (although data were missing in 18 % of patients) versus 73 % in our study. In interpreting these results, and comparing them with our figures, one must account for the differences that exist between countries in organization of post-ICU care for patients dependent on mechanical ventilation or other forms of advanced care due to chronic critical illness. Weaning centers and long-term care hospitals do not exist in Belgium as separate institutions, but are embedded within ICUs and pulmonology departments certified for providing home mechanical ventilation. As such, part of the differences in outcome between the US study and ours are probably related to the fact that long-term care hospitals take over part of the weaning process in difficult-to-wean patients from the ICU and admit patients from ICUs in an earlier stage of their critical illness: consequently, both in-hospital mortality and weaning success are higher than in our cohort, which was a more selected group of patients that was offered prolonged ventilation after failing a more extended weaning effort at the ICU.

Very few data exist on QOL in patients with prolonged or chronic mechanical ventilation following acute critical illness. In the Ventilation Outcomes Study, functional status (as measured by Zubrod Functional Status Scores) in LTAC hospital survivors was good (ambulatory and self-care possible, or better) in 60 % and poor (bedridden at least 50 % of time and no self-care) in the remaining 40 % [13]. Comparison with our data is difficult given the different QOL scores used. No data on mental health were provided in the Ventilation Outcomes Study.

The SRI questionnaire was originally conceived to measure QOL in patients requiring chronic non-invasive home ventilation [15]. In a multicenter study with 85 patients requiring non-invasive home ventilation [16], the summary score of SRI was 61 + −15, which is 10 points higher than in our population; a similar difference can be seen in the physical component summary of the SF-36. On the other hand, the mental component summary score was similar in our patients. The better physical QOL in patients receiving non-invasive home ventilation may reflect the absence of a tracheostomy and its interference with speech and eating, but also differences in the path leading to chronic respiratory failure. In our patient population, the acute critical illness and its subsequent prolonged ICU stay may have caused additional functional and cognitive impairments that have been grouped under the moniker of ‘chronic critical illness’ [17, 18].

In our study, QOL measurements in domains reflecting emotional and social functioning were better than QOL in domains representing physical functioning, and were not different between ventilator-dependent and weaned patients. One elegant definition of overall QOL is to regard it as the interaction of human needs and the subjective perception of their fulfillment. Patients may accept conditions of dependency and physical limitation as long as their perceived role in social relations and life experiences in general remain substantial and meaningful. Interestingly, in the aforementioned multicenter study of QOL in patients requiring home noninvasive mechanical ventilation, psychological well-being and social functioning improved between 1 month and 1 year following the start of ventilation in a number of subgroups of patients [16]. In our study, we only measured QOL once after a median of approximately 4 to 5 years after ICU discharge. It is conceivable that the relatively long time interval between the ICU admission and QOL evaluated has permitted some form of mental adaptation and allowed the patients to come to terms with the chronic but stable situation of dependency. Overall SF-36 scores of the patients included in the present study are in the range of what is observed in patients with chronic diseases as severe renal failure or heart failure in NYHA III and IV classes [5, 19].

Limitations of the study are its monocentric and retrospective design, which may limit generalization. Post-ICU care is little structured in Belgium, and results may depend on local expertise. The studied cohort was highly heterogeneous in terms of comorbidity and cause of acute respiratory failure and no discriminative sets of criteria that identify a good candidate for post-ICU mechanical ventilation can be deduced. Patients with underlying neuromuscular disease are more prevalent in the chronically ventilated group: the fact that these patients experienced a slowly progressive disease course prior to the ARF event may have favored their acceptance of the chronic mechanical ventilation.

QOL assessment was done in all patients within a single fixed time frame, and the time between ICU discharge and QOL assessment was variable for the individual patient. The patient numbers are too small to allow meaningful subgroup analysis. No QOL measurement is available for the period before ICU admission, but this is a limitation present in most studies addressing post-ICU outcomes. As such, it is difficult to distinguish the contribution of chronic ventilation in patients whose QOL may have been severely affected by the presence of severe underlying comorbidities. Survival bias may be of concern, as QOL only was assessed in patients surviving until 2014; QOL in patients deceased before this date is likely to have been lower. Finally, the SRI questionnaire has been developed in German language but has not been validated for the Dutch translation.

Our study is however one of the few providing long-term outcome data in patients who fail to wean from invasive mechanical ventilation in the ICU. With further advances in critical care and with the accumulation of comorbidities in an ageing population, this particular patient group is very likely to expand and to impose an increasing burden on our ICUs and overall healthcare delivery. Despite the limitations mentioned above, our data show that at least in selected patients, mechanical ventilation prolonged outside the ICU may be a valuable treatment, both in terms of survival and perceived QOL. However, given the intrinsic limitations of this retrospective and monocentric study, these results should be considered as preliminary, urging for prospective and preferentially multicenter studies to confirm them.

## Conclusion

Long-term survival in patients with tracheostomy selected for prolonging mechanical ventilation outside the ICU after failure to wean was not significantly different from that of patients with tracheostomy who were weaned from mechanical ventilation in the ICU. Despite the physical QOL scores being low in both groups, mental QOL was acceptable.

## Abbreviations

ARF: Acute Respiratory Failure
ICU: Intensive Care Unit
MICU: Medical Intensive Care Unit
PF: Physical functioning
QOL: Quality of life
SF: Social functioning
SF-36: Medical Outcomes Study 36-item Short Form Health Survey
SRI: Severe Respiratory Insufficiency
